# Crystal structure of 3-bromo­pyridine *N*-oxide

**DOI:** 10.1107/S205698901501909X

**Published:** 2015-10-24

**Authors:** Matthew G. Hutchinson, Will E. Lynch, Clifford W. Padgett

**Affiliations:** a Department of Chemistry and Physics, Armstrong State University, Savannah, GA 31419, USA

**Keywords:** crystal structure, 3-bromo­pyridine *N*-oxide, herringbone pattern

## Abstract

In the title compound, C_5_H_4_BrNO, there are two mol­ecules in the asymmetric unit that are related by a pseudo-inversion center. The two independent mol­ecules are approximately planar, with an observed (ring–ring) angle of 5.49 (13)°. The crystal structure exhibits a herringbone pattern with the zigzag running along the *b-*axis direction. The least-squares plane containing the rings of both asymmetric molecules and the plane containing the symmetrically related mol­ecules make a plane–plane angle of 66.69 (10)°, which makes the bend of the herringbone pattern. The bromo group on one mol­ecule points to the bromo group on the neighboring mol­ecule, with a Br⋯Br inter­molecular distance of 4.0408 (16) Å. The herringbone layer-to-layer distance is 3.431 (4) Å with a shift of 1.742 (7) Å. There are no short contacts, hydrogen bonds, or π–π inter­actions.

## Related literature   

For the synthesis of pyridine *N*-oxide-related compounds, see: Rousseau & Robins (1965 ▸). For an example of the chemistry of the title compound and its use in catalysed cyclization of alkynyl oxiranes and oxetanes, see: Gronnier *et al.* (2012 ▸).
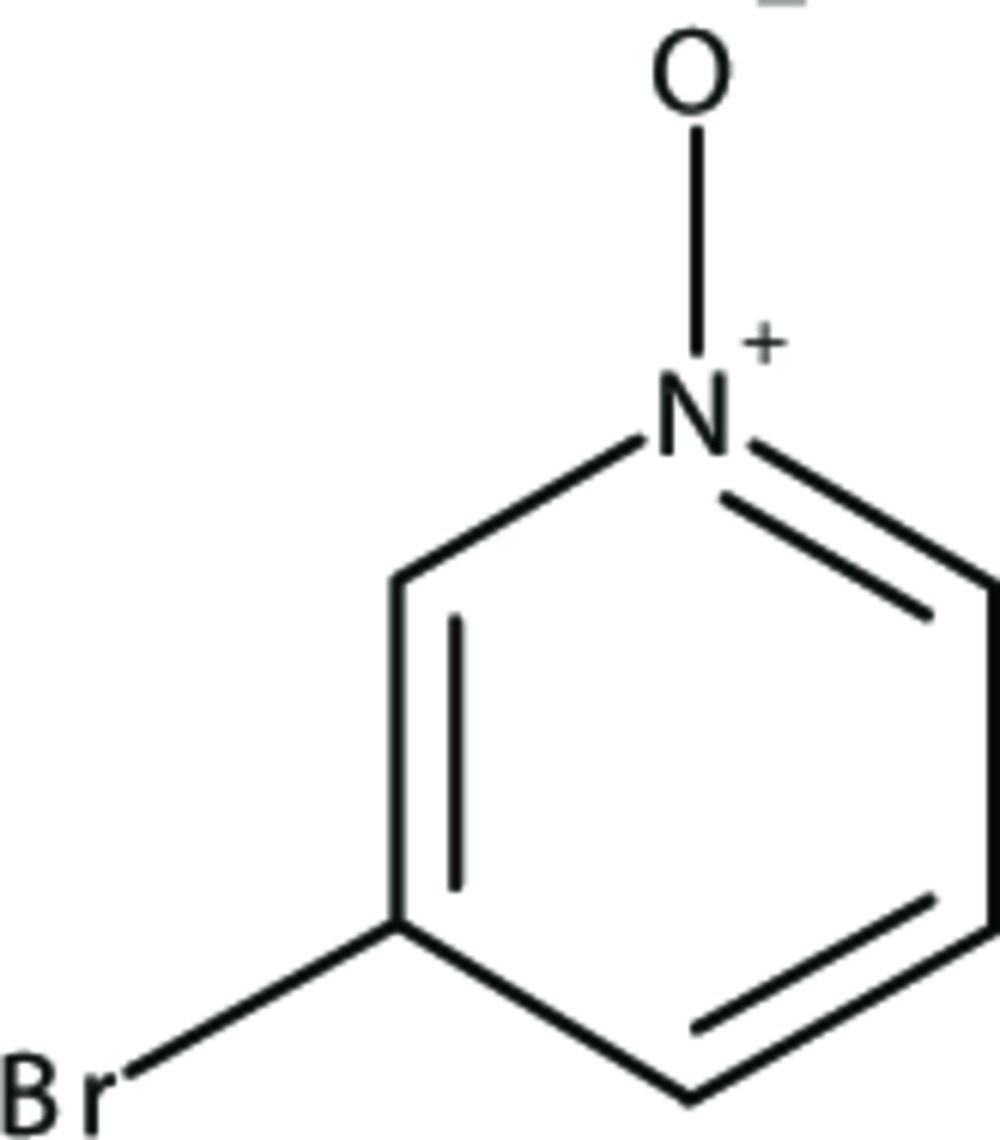


## Experimental   

### Crystal data  

C_5_H_4_BrNO
*M* *_r_* = 174.00
Monoclinic, 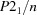

*a* = 7.832 (5) Å
*b* = 18.398 (10) Å
*c* = 8.298 (5) Å
β = 92.906 (5)°
*V* = 1194.2 (12) Å^3^
*Z* = 8
Mo *K*α radiation
μ = 6.77 mm^−1^
*T* = 173 K
0.3 × 0.3 × 0.2 mm

### Data collection  

Rigaku XtaLAB mini diffractometer
Absorption correction: multi-scan (*REQAB*; Rigaku, 1998 ▸) *T*_min_ = 0.189, *T*_max_ = 0.257
12561 measured reflections
2732 independent reflections
1881 reflections with *I* > 2σ(*I*)
*R*_int_ = 0.056

### Refinement  

*R*[*F* ^2^ > 2σ(*F* ^2^)] = 0.041
*wR*(*F* ^2^) = 0.092
*S* = 1.09
2732 reflections
146 parameters
H-atom parameters constrained
Δρ_max_ = 0.50 e Å^−3^
Δρ_min_ = −0.67 e Å^−3^

### 

Data collection: *CrystalClear-SM Expert* (Rigaku, 2011 ▸); cell refinement: *CrystalClear-SM Expert*; data reduction: *CrystalClear-SM Expert*; program(s) used to solve structure: *SHELXT* (Sheldrick, 2015*a* ▸); program(s) used to refine structure: *SHELXL2014* (Sheldrick, 2015*b* ▸); molecular graphics: *OLEX2* (Dolomanov *et al.*, 2009 ▸); software used to prepare material for publication: *OLEX2*.

## Supplementary Material

Crystal structure: contains datablock(s) I. DOI: 10.1107/S205698901501909X/lh5792sup1.cif

Structure factors: contains datablock(s) I. DOI: 10.1107/S205698901501909X/lh5792Isup2.hkl

Supporting information file. DOI: 10.1107/S205698901501909X/lh5792Isup3.cml

**Fig. 1.**
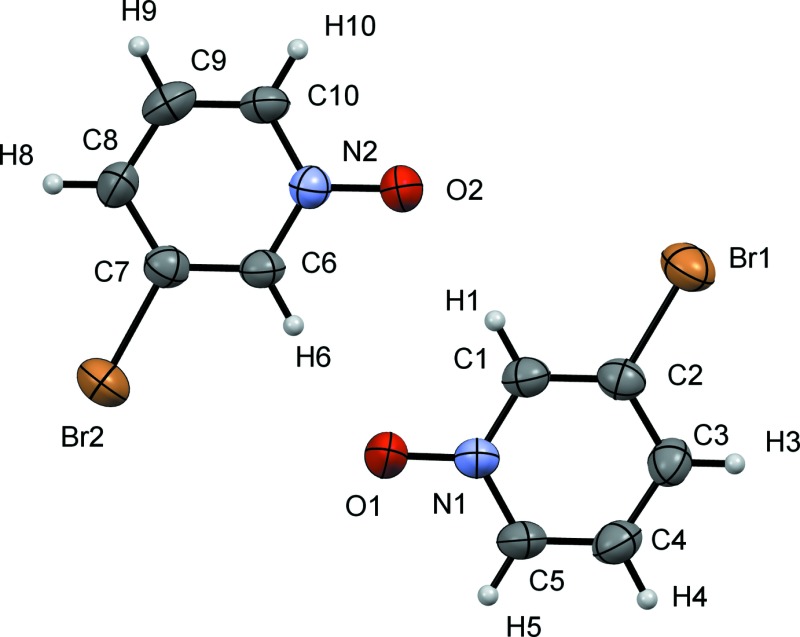
The asymmetric unit of the title compound. Displacement ellipsoids are drawn at the 50% probability level.

. DOI: 10.1107/S205698901501909X/lh5792fig1.tif
The asymmetric unit of the title compound. Displacement ellipsoids are drawn at the 50% probability level.

CCDC reference: 1430552

Additional supporting information:  crystallographic information; 3D view; checkCIF report

## supplementary crystallographic information

### S1. Structural commentary

Details of the synthesis pyridine N-oxide related compounds appears in the literature Rousseau & Robins (1965). The title compound is used in the catalyzed cyclization of alkynyl oxiranes and oxetanes Gronnier, *et al.* (2012). The asymmetric unit of the title compound is shown in Fig. 1. There are two molecules in the asymmetric unit that are related by a pseudo-inversion center. The inversion symmetry is broken by the nonplanar arrangement of the two molecules. The two independent molecules are found to be nearly planar with an observed twist angle of 5.49 (13)° and a fold angle of 1.40 (13)°. The distance between O2···Br1 is 4.307 (3) Å and the distance between O1···Br2 is 4.196 (3) Å. The structure exhibits a herringbone pattern with the zigzag running along the b axis. The least-squares plane containing both rings of the asymmetric unit and the plane containing the symmetrically-related molecules have a plane-plane angle of 66.69 (10)°, which makes the bend of the herringbone pattern. The bromo group on one molecule points to the bromo group on the neighboring molecule with the Br1···Br2 inter­molecular distance at 4.0408 (16) Å. The herringbone layer-to-layer distance is 3.431 (4) Å with a shift of 1.742 (7) Å.

### S2. Synthesis and crystallization

3-Bromo­pyridine *N*-oxide was purchased from Sigma-Aldrich and 0.10 g was dissolved in approximately 50 mL of methanol. Diffraction quality crystals were obtained by slow evaporation of the solvent.

### S3. Refinement

H atoms were placed in calculated positions with C–H = 0.93Å and U_iso_(H) = 1.2U_eq_(C).

### Crystal data

**Table:**

C_5_H_4_BrNO	*F*(000) = 672
*M**_r_* = 174.00	*D*_x_ = 1.936 Mg m^−^^3^
Monoclinic, *P*2_1_/*n*	Mo *K*α radiation, λ = 0.71073 Å
*a* = 7.832 (5) Å	Cell parameters from 2269 reflections
*b* = 18.398 (10) Å	θ = 1.6–27.4°
*c* = 8.298 (5) Å	µ = 6.77 mm^−^^1^
β = 92.906 (5)°	*T* = 173 K
*V* = 1194.2 (12) Å^3^	Prism, colorless
*Z* = 8	0.3 × 0.3 × 0.2 mm

### Data collection

**Table:**

Rigaku XtaLAB mini diffractometer	2732 independent reflections
Radiation source: Sealed Tube	1881 reflections with *I* > 2σ(*I*)
Graphite Monochromator monochromator	*R*_int_ = 0.056
Detector resolution: 13.6612 pixels mm^-1^	θ_max_ = 27.5°, θ_min_ = 2.7°
ω scans	*h* = −10→10
Absorption correction: multi-scan (*REQAB*; Rigaku, 1998)	*k* = −23→23
*T*_min_ = 0.189, *T*_max_ = 0.257	*l* = −10→10
12561 measured reflections	

### Refinement

**Table:**

Refinement on *F*^2^	Hydrogen site location: inferred from neighbouring sites
Least-squares matrix: full	H-atom parameters constrained
*R*[*F*^2^ > 2σ(*F*^2^)] = 0.041	*w* = 1/[σ^2^(*F*_o_^2^) + (0.0225*P*)^2^ + 1.184*P*] where *P* = (*F*_o_^2^ + 2*F*_c_^2^)/3
*wR*(*F*^2^) = 0.092	(Δ/σ)_max_ < 0.001
*S* = 1.09	Δρ_max_ = 0.50 e Å^−^^3^
2732 reflections	Δρ_min_ = −0.67 e Å^−^^3^
146 parameters	Extinction correction: *SHELXL2014* (Sheldrick, 2015b), Fc^*^=kFc[1+0.001xFc^2^λ^3^/sin(2θ)]^-1/4^
0 restraints	Extinction coefficient: 0.0059 (6)
Primary atom site location: structure-invariant direct methods	

### Special details

**Table:**

Geometry. All e.s.d.'s (except the e.s.d. in the dihedral angle between two l.s. planes) are estimated using the full covariance matrix. The cell e.s.d.'s are taken into account individually in the estimation of e.s.d.'s in distances, angles and torsion angles; correlations between e.s.d.'s in cell parameters are only used when they are defined by crystal symmetry. An approximate (isotropic) treatment of cell e.s.d.'s is used for estimating e.s.d.'s involving l.s. planes.

### Fractional atomic coordinates and isotropic or equivalent isotropic displacement parameters (Å^2^)

**Table:**

	*x*	*y*	*z*	*U*_iso_*/*U*_eq_	
Br2	0.67164 (6)	0.60185 (3)	0.12441 (6)	0.0635 (2)	
Br1	0.49425 (6)	0.20942 (3)	0.64430 (6)	0.0639 (2)	
O2	0.3319 (3)	0.40963 (15)	0.4152 (4)	0.0529 (8)	
N2	0.3246 (4)	0.46580 (17)	0.3178 (4)	0.0405 (7)	
O1	0.8214 (4)	0.41613 (18)	0.3888 (4)	0.0688 (10)	
N1	0.8315 (4)	0.36058 (18)	0.4861 (4)	0.0458 (8)	
C6	0.4715 (5)	0.4993 (2)	0.2786 (4)	0.0399 (9)	
H6	0.5765	0.4832	0.3222	0.048*	
C2	0.6948 (5)	0.2634 (2)	0.6143 (4)	0.0423 (9)	
C1	0.6861 (5)	0.3230 (2)	0.5159 (5)	0.0439 (9)	
H1	0.5814	0.3380	0.4694	0.053*	
C7	0.4632 (5)	0.5572 (2)	0.1741 (5)	0.0419 (9)	
C3	0.8487 (6)	0.2403 (2)	0.6878 (5)	0.0494 (10)	
H3	0.8548	0.1997	0.7547	0.059*	
C10	0.1717 (5)	0.4901 (2)	0.2572 (5)	0.0473 (10)	
H10	0.0713	0.4681	0.2873	0.057*	
C5	0.9832 (5)	0.3399 (2)	0.5567 (5)	0.0500 (10)	
H5	1.0818	0.3659	0.5372	0.060*	
C4	0.9919 (5)	0.2807 (2)	0.6567 (5)	0.0519 (11)	
H4	1.0970	0.2673	0.7049	0.062*	
C8	0.3107 (5)	0.5830 (2)	0.1079 (5)	0.0525 (11)	
H8	0.3062	0.6223	0.0375	0.063*	
C9	0.1645 (5)	0.5475 (3)	0.1510 (5)	0.0571 (12)	
H9	0.0589	0.5628	0.1073	0.069*	

### Atomic displacement parameters (Å^2^)

**Table:**

	*U*^11^	*U*^22^	*U*^33^	*U*^12^	*U*^13^	*U*^23^
Br2	0.0504 (3)	0.0630 (3)	0.0777 (4)	−0.0142 (2)	0.0089 (2)	0.0102 (2)
Br1	0.0534 (3)	0.0706 (4)	0.0674 (3)	−0.0198 (2)	0.0011 (2)	0.0044 (2)
O2	0.0458 (17)	0.0450 (17)	0.0679 (19)	−0.0009 (13)	0.0033 (15)	0.0144 (14)
N2	0.0385 (19)	0.0358 (18)	0.0471 (18)	0.0008 (14)	0.0014 (15)	0.0007 (14)
O1	0.0456 (19)	0.072 (2)	0.089 (2)	0.0061 (15)	0.0075 (17)	0.0410 (18)
N1	0.0341 (18)	0.049 (2)	0.055 (2)	0.0025 (15)	0.0058 (16)	0.0087 (17)
C6	0.030 (2)	0.044 (2)	0.046 (2)	−0.0008 (16)	−0.0014 (17)	−0.0017 (17)
C2	0.039 (2)	0.047 (2)	0.041 (2)	−0.0045 (18)	0.0027 (18)	−0.0059 (18)
C1	0.034 (2)	0.052 (3)	0.046 (2)	0.0011 (18)	−0.0014 (17)	−0.0006 (19)
C7	0.039 (2)	0.040 (2)	0.047 (2)	−0.0015 (17)	0.0039 (18)	−0.0035 (18)
C3	0.053 (3)	0.046 (2)	0.048 (2)	−0.002 (2)	−0.009 (2)	0.0049 (19)
C10	0.028 (2)	0.054 (3)	0.060 (3)	0.0035 (18)	0.0047 (19)	0.001 (2)
C5	0.030 (2)	0.061 (3)	0.059 (3)	0.0003 (19)	0.0000 (19)	0.006 (2)
C4	0.041 (2)	0.055 (3)	0.058 (3)	0.006 (2)	−0.008 (2)	0.001 (2)
C8	0.046 (3)	0.052 (3)	0.059 (3)	0.010 (2)	0.003 (2)	0.018 (2)
C9	0.039 (2)	0.064 (3)	0.067 (3)	0.014 (2)	−0.006 (2)	0.004 (2)

### Geometric parameters (Å, º)

**Table:**

Br2—C7	1.892 (4)	C1—H1	0.9300
Br1—C2	1.885 (4)	C7—C8	1.373 (5)
O2—N2	1.312 (4)	C3—H3	0.9300
N2—C6	1.359 (5)	C3—C4	1.381 (6)
N2—C10	1.351 (5)	C10—H10	0.9300
O1—N1	1.302 (4)	C10—C9	1.374 (6)
N1—C1	1.365 (5)	C5—H5	0.9300
N1—C5	1.352 (5)	C5—C4	1.369 (6)
C6—H6	0.9300	C4—H4	0.9300
C6—C7	1.373 (5)	C8—H8	0.9300
C2—C1	1.367 (5)	C8—C9	1.380 (6)
C2—C3	1.390 (5)	C9—H9	0.9300
			
O2—N2—C6	119.6 (3)	C2—C3—H3	121.7
O2—N2—C10	120.0 (3)	C4—C3—C2	116.7 (4)
C10—N2—C6	120.4 (3)	C4—C3—H3	121.7
O1—N1—C1	119.0 (3)	N2—C10—H10	120.0
O1—N1—C5	120.9 (3)	N2—C10—C9	120.0 (4)
C5—N1—C1	120.1 (3)	C9—C10—H10	120.0
N2—C6—H6	120.4	N1—C5—H5	119.9
N2—C6—C7	119.2 (3)	N1—C5—C4	120.2 (4)
C7—C6—H6	120.4	C4—C5—H5	119.9
C1—C2—Br1	119.0 (3)	C3—C4—H4	119.2
C1—C2—C3	121.5 (4)	C5—C4—C3	121.7 (4)
C3—C2—Br1	119.4 (3)	C5—C4—H4	119.2
N1—C1—C2	119.9 (4)	C7—C8—H8	121.7
N1—C1—H1	120.1	C7—C8—C9	116.7 (4)
C2—C1—H1	120.1	C9—C8—H8	121.7
C6—C7—Br2	117.4 (3)	C10—C9—C8	121.4 (4)
C6—C7—C8	122.3 (4)	C10—C9—H9	119.3
C8—C7—Br2	120.3 (3)	C8—C9—H9	119.3
			
Br2—C7—C8—C9	−179.7 (3)	N1—C5—C4—C3	0.4 (7)
Br1—C2—C1—N1	−176.6 (3)	C6—N2—C10—C9	2.2 (6)
Br1—C2—C3—C4	177.8 (3)	C6—C7—C8—C9	−0.3 (6)
O2—N2—C6—C7	178.8 (3)	C2—C3—C4—C5	−0.8 (6)
O2—N2—C10—C9	−177.9 (4)	C1—N1—C5—C4	0.8 (6)
N2—C6—C7—Br2	179.8 (3)	C1—C2—C3—C4	0.1 (6)
N2—C6—C7—C8	0.4 (6)	C7—C8—C9—C10	1.1 (7)
N2—C10—C9—C8	−2.1 (7)	C3—C2—C1—N1	1.1 (6)
O1—N1—C1—C2	178.3 (4)	C10—N2—C6—C7	−1.4 (5)
O1—N1—C5—C4	−179.0 (4)	C5—N1—C1—C2	−1.6 (6)
