# Supplementary figures and images for: Crystal structure of 3-bromo­pyridine N-oxide
Source: Acta Crystallogr E Crystallogr Commun. 2015 Oct 24;71(Pt 11):o869. doi: 10.1107/S205698901501909X (PMC4645004; doi:10.1107/S205698901501909X)

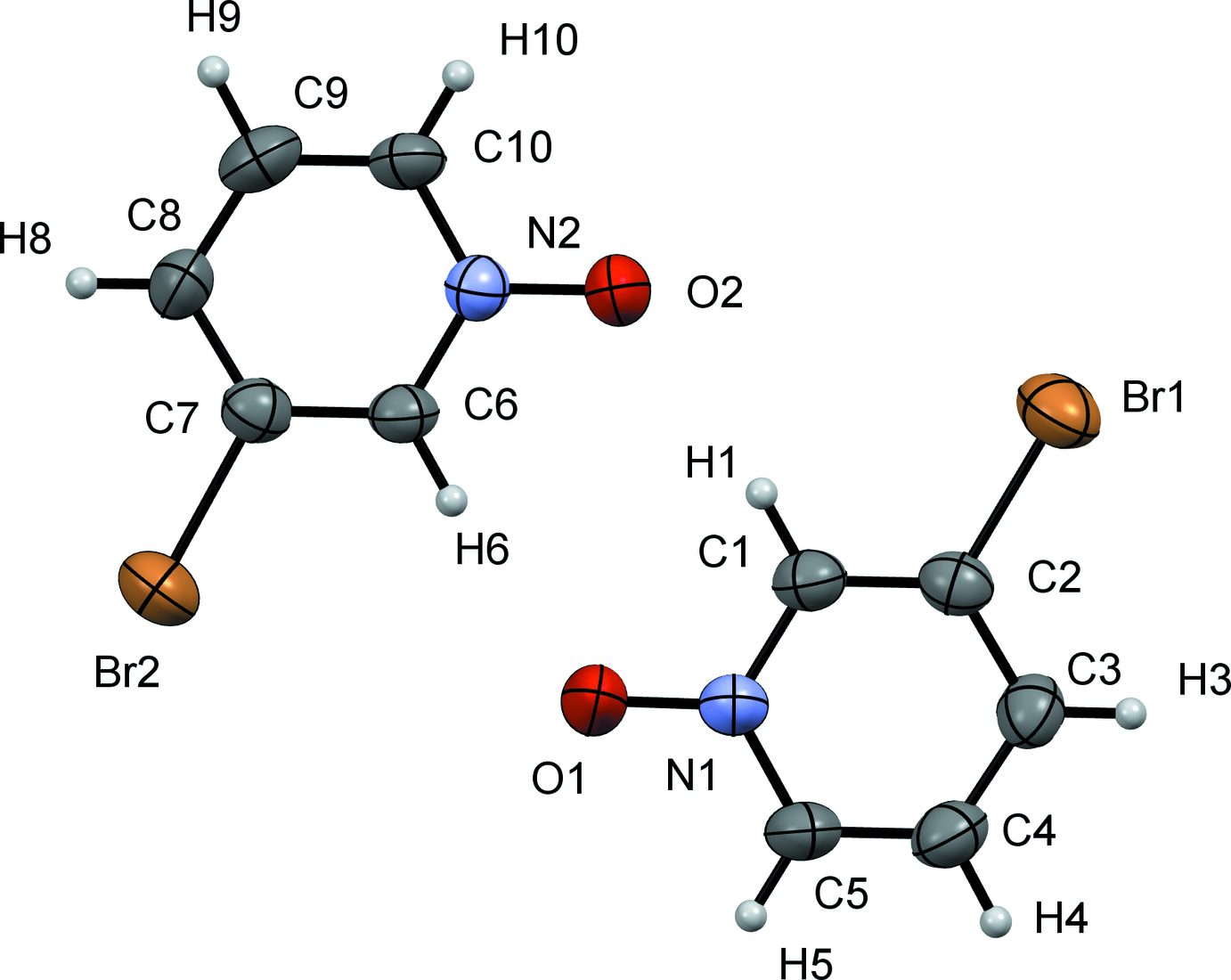

Supplement: Supplementary file 4 [file e-71-0o869-fig1.tif]
